# Structure and properties of slow-resorbing nanofibers obtained by (co-axial) electrospinning as tissue scaffolds in regenerative medicine

**DOI:** 10.7717/peerj.4125

**Published:** 2017-12-18

**Authors:** Andrzej Hudecki, Joanna Gola, Saeid Ghavami, Magdalena Skonieczna, Jarosław Markowski, Wirginia Likus, Magdalena Lewandowska, Wojciech Maziarz, Marek J. Los

**Affiliations:** 1 Institute of Nonferrous Metals, Gliwice, Poland; 2 Department of Molecular Biology, School of Pharmacy with the Division of Laboratory Medicine in Sosnowiec, Medical University of Silesia, Sosnowiec, Poland; 3 Department of Human Anatomy and Cell Science, University of Manitoba, Winnipeg, MB, Canada; 4 Health Policy Research Center, Institute of Health Shiraz University of Medical Sciences, Shiraz, Iran; 5 Biosystems Group, Institute of Automatic Control, Faculty of Automatics, Electronics and Informatics, and Biotechnology Centre, Silesian University of Technology, Gliwice, Poland; 6 ENT Department, School of Medicine in Katowice, Medical University of Silesia in Katowice, Katowice, Poland; 7 Department of Anatomy, School of Health Sciences in Katowice, Medical University of Silesia, Katowice, Poland; 8 Department of Pathology, Pomeranian Medical University, Szczecin, Poland; 9 Institute of Metallurgy and Material Science Polish Academy of Sciences, Kraków, Poland; 10 Małopolska Center of Biotechnology, Kraków, Poland; 11 Linkocare Life Sciences AB, Linkoping, Sweden; 12 Centre de biophysique moléculaire CNRS, Rue Charles Sadron, Orleans cedex 2, France

**Keywords:** Policaprolacotne, Nanofibers, Solution electrospinnning, Core-shell nanofibers, Co-axial electrospinning

## Abstract

With the rapid advancement of regenerative medicine technologies, there is an urgent need for the development of new, cell-friendly techniques for obtaining nanofibers—the raw material for an artificial extracellular matrix production. We investigated the structure and properties of PCL_10_ nanofibers, PCL_5_/PCL_10_ core-shell type nanofibers, as well as PCL_5_/PCL_Ag_ nanofibres prepared by electrospinning. For the production of the fiber variants, a 5–10% solution of polycaprolactone (PCL) (M_w_ = 70,000–90,000), dissolved in a mixture of formic acid and acetic acid at a ratio of 70:30 m/m was used. In order to obtain fibers containing PCL_Ag_ 1% of silver nanoparticles was added. The electrospin was conducted using the above-described solutions at the electrostatic field. The subsequent bio-analysis shows that synthesis of core-shell nanofibers PCL_5_/PCL_10_, and the silver-doped variant nanofiber core shell PCL_5_/PCL_Ag_, by using organic acids as solvents, is a robust technique. Furthermore, the incorporation of silver nanoparticles into PCL_5_/PCL_Ag_ makes such nanofibers toxic to model microbes without compromising its biocompatibility. Nanofibers obtained such way may then be used in regenerative medicine, for the preparation of extracellular scaffolds: (i) for controlled bone regeneration due to the long decay time of the PCL, (ii) as bioscaffolds for generation of other types of artificial tissues, (iii) and as carriers of nanocapsules for local drug delivery. Furthermore, the used solvents are significantly less toxic than the solvents for polycaprolactone currently commonly used in electrospin, like for example chloroform (CHCl_3_), methanol (CH_3_OH), dimethylformamide (C_3_H_7_NO) or tetrahydrofuran (C_4_H_8_O), hence the presented here electrospin technique may allow for the production of multilayer nanofibres more suitable for the use in medical field.

## Introduction

In simple terms, tissues are composed of cells and extracellular matrix (ECM). Rapid development of regenerative medicine techniques has prompted the development of technologies for the generation of given types of cells on demand, i.e., by reprogramming with subsequent differentiation, or by transdifferentiation (Cieslar-Pobuda et al., 2016). To complete the production of artificial tissues, one also needs ECM that would give the cells proper support, and maintain appropriate stiffness/elasticity and strength of the manufactured tissue-replacements. Beside natural compounds such as collagens, proteoglycans and glycoproteins, artificial nanofibers become increasingly tested as replacement-components of artificial extracellular matrices (O’Brien, 2011). Although several types of nanofibers have been manufactured so far, none of them entered broadly into clinical practice beside the experimental phase, mainly due to the biocompatibility issues (Sperling et al., 2016).

**Figure 1:**
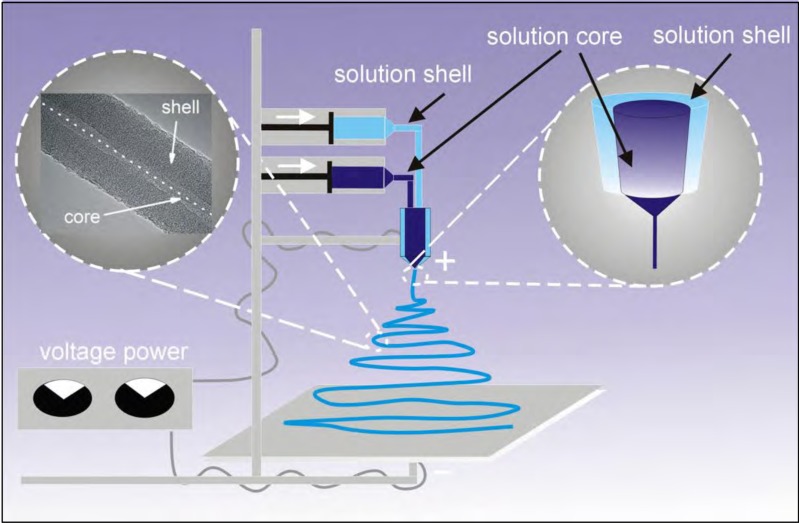
Diagram of the applied coaxial electrospinning process.

The preparation of micro- and nanofibers under an electrostatic field could be performed either by electrospin from the raw material in solution (Fig. 1) or from the melted material. The initial properties of the polymeric material will dictate what kind of method for the preparation of the nanofibers will be chosen. For example, in case of natural polymers like chitosan, which do not melt at higher temperature, electrospinning is chosen for the preparation of micro- and nanofibers (Cai et al., 2010; Paneva et al., 2009). Melt electrospinning is chosen for the generation of nanofibres which form thermoplastic polymers such as polypropylene, which is difficult to dissolve but melts at higher temperatures (Fang et al., 2012). Some polymeric materials could be converted into the nanofiber using electrospinning of the solution and also melt-electrospinning. Examples of these types of materials serve PVA, N6 (Brown et al., 2014), PMMA (Qian et al., 2010).

Electrospinning is the most common method for the preparation of nanofibres from the solution. The properties of the nanofibers obtained in the process, like diameter of fibers, surface morphology, and regularity of shape depend on three main conditions: (i) environment (the temperature of gas in which electrospinning occurs, gas humidity, gas flow velocity), (ii) process conditions (i.e., the type of electrodes used, the flow rate of the solution, the distance between the electrodes, the rotational speed of the rotary collector), and (iii) the properties of the dissolved material in solution, which is the sum of the properties of the polymeric material, the properties of the solvent, and the properties of introduced additives. Each of these three sets of conditions and properties is directly or indirectly related to the others (Andrady, 2008). For example, the properties of the polymeric material will dictate the type of solvent, which is used to dissolve it (Wang et al., 2010). Because most polymers are dielectrics, adding the solvent will decrease the conductivity of the solution. However, there are polymeric materials, such as chitosan, which when added to the solution, will increase the conductivity of the resulting solution. Converting the polymer solution into nanofibres occurs due to the evaporation of the used solvent.

A variation of the electrospinning process called coaxial-electrospinning was employed in this paper (Fig. 1) (Zhang et al., 2010). Just like during the standard electrospinning process, shortly after passing the nozzle, under optimal conditions, a Taylor cone is created (double cone in coaxial electrospin). The shape and properties of Taylor cones depend on the characteristics of the respected polymer-forming solutions. At the contact-zone both solutions interact, and the nature of this interaction is formative for the coaxial electrospinning process. The coating solution is stretched by a electrostatic field within the zone of straight-forward flow (according to Faraday’s law, the charge accumulates on the outer wall of a structure). The flow of the core-forming solution is also stretched due to the interactions and friction imposed on it by the coating solution. The shaping (morphology) of nanofibers of the core-shell polymer-type is also strongly influenced by the evaporation rate of solvents used in the process. If the core-forming material is dissolved with a solvent with a much higher evaporation rate than the solvent in the coating polymer, a thin layer (wall) will form within the core that will cause the solvent to diffuse slowly. In effect, an under-pressure is created within the core, which will cause the collapse of the core, and results in a strip/ribbon shape of the nanofibre.

Coaxial electrospinning technology is significantly more difficult than the single-material electrospining; however, it enables the preparation of fibers having much broader applications. For example, it allows for obtaining hollow fibers (Khajavi & Abbasipour, 2012; Kroczak et al., 2006; Srivastava et al., 2007), core–shell type fibers (Yu et al., 2012), encapsulating other compounds and biologic materials (McCann, Marquez & Xia, 2006), encapsulation of macromolecular materials such as i.e., DNA, preparation of composite fibers (Xu et al., 2004), a combination of polymeric materials with different properties (Zhang et al., 2010), or production of tissue scaffolding with a desired disintegration time (Szabo et al., 2014). The purpose of the following study was to prepare core–shell nanofibers using a combination of two organic acids as solvents for PCL. Organic acids at trace-quantities are, in general, better tolerated by living tissues than organic solvents commonly used for electrospinning.

## Materials and Methods

### Materials

For the preparation of a biodegradable composite fiber we used PCL having a molecular weight of *M*_*w*_ = 70,000–90,000; Sigma Aldrich, St. Louis, MO, USA. We used organic solvents: formic acid (99.9% purity), and acetic acid (also 99.9% purity) purchased from Sigma Aldrich (St. Louis, MO, USA). Silver nanoparticles with a diameter of 20–30 nm, and the purity of 95.95% were from Skyspring Nanomaterials.

### Dissolving the polymers

For the preparation of nanofibres applied:

(a) a mixture of formic acid and acetic acid in a ratio of 70:30 m/m was prepared, to which the silver nanoparticles were introduced in an amount of 1%, while using a ultrasonic homogenizer for 30 min. Next, to the solution PCL (*M*_*w*_ = 70,000–90,000) was added, and dissolved, to give a 10% solution of PCL_Ag_
(b) a mixture of formic acid and acetic acid in a ratio of 70:30 m/m was prepared, to which the granules of PCL (*M*_*w*_ = 70,000–90,000) was added while stirring with the magnetic stirrer, until the 10% solution of PCL was obtained (PCL_10_). Following a similar approach, also the PCL_5_ solution was prepared
(c) using a similar approach, we also prepared PCL_5_ solution. The 70:30 mixture-ration for both solvents was determined empirically as a good compromise of material solubility, and evaporation ratio.

### Electrospinning

The PCL_5_-, PCL_10_-, and PCL_∕Ag_-solutions (prepared as described above) were used. For the preparation of solid (one layer) nanofibres the PCL_10_ was used. The PCL_10_ solution was combined with PCL_5_ solution to obtain a core–shell nanofibers PCL_5_/PCL_10_. PCL_5_ solution was combined with the PCL_∕Ag_ solution to prepare the PCL_5_/PCL_∕Ag_ nanofibres. The electrospin-parameters were as follow: the flow range 0.05–1.5 ml/h, the voltage range 0.8–1.2 kV/cm, collector (negative electrode): SS Flat Plate (400 mm × 400 mm), single nozzle injector (positive electrode) outer needle 0.9 mm, co-axial injector (positive electrode) inner needle 0,6 mm, outer needle 1,4 mm, humidity: 32% ± 4%, temperature: 23 °C ± 2 °C. The electrospin was done using Coaxial Electrospinning & Electrospray 550 from Yflow.

### Testing of the obtained nanofibers

#### FTIR

The evaluation of the structure of PCL was performed using FTIR infrared spectroscopy. The technique is used to determine the presence of functional groups within PCL. For visualization purpose, the sample was combined with KBr powder in a proportion 0.1 mg of fibers to 3g KBr, compressed to form pellets and then placed in a desiccator with a moisture binder (sillica gel) for 30 min at 40 °C. After drying, the samples were placed in the autosampler of the device, and were scanned 128 times with a resolution of 4 cm^−1^ in wavelength range between 400–4,000 cm^−1^.

#### XRD

X-ray diffraction techniques were used to determine the structure of silver nanoparticles in PCL_5_/PCL_∕Ag_ nanofibres. The X-ray diffraction measurements for selected samples were Performed at ambient temperature using a Rigaku MiniFlex 600 Diffractometer (Rigaku Corporation, Tokyo, Japan) with Cu Kα radiation (λ = 1.5406 Å), at tube voltage of 40 kV and a current of 15 mA using a D/teX Ultra silicon strip detector.

#### SEM & TEM

The topography of the obtained nanofibres were analyzed by using scanning electron microscope Zeiss Supra at different settings of the acceleration voltage and magnification ranges, selected for optimal observation of samples. Samples of the tested nanofibres (PCL_10_, PCL_5_/PCL_10_, PCL_5_/PCL_Ag_) were applied directly onto the surface of copper mesh and subjected to structural analysis using a high resolution transmission electron microscope TEM at an accelerating voltage of 300 kV and modes of the Fourier transformation FFT inverse Fourier transformation IFFT, using a bright-field detector BF, a high resolution wide angle dark field detector HAADF, STEM scan mode transmission, and a standard EDS detector.

#### Cell viability tests

The biocompatibility (potential toxicity) of the generated nanofibres was assessed by MTT assay using normal human dermal fibroblasts (NHDF) obtained from Professor G. Kratz, (Cieslar-Pobuda et al., 2014), or from Clonetics (CC-2511; San Diego, CA, USA). NHDF were used because they are common starting material for reprogramming or transdifferentiation techniques, both commonly used for the production of desired cell types for regenerative medicine purposes. The procedures were performed similarly as described previously (Magnusson et al., 2015). The cells were cultured in DMEM-F12 (SIGMA) supplemented with 10% FBS fetal calf serum solution (SIGMA), in sterile and standard conditions (37 °C, 60% humidity, 5% CO_2_), while being kept in logarythmic phase. For testing, cells were trypsinized, and plated on the test composites placed in 96-well plates (final density suspend 2 × 10^4^ cells/well). Following 72 h incubation, MTT-reagent (3–(4,5-dimethylthiazol-2-yl)-2,5-diphenyltetrazolium bromide, Sigma-Aldrich) was added at concentration 0.5 mg/ml. After further 4 h incubation, the MTT solution was removed and the formazan product was dissolved in dimethyl sulfoxide. The absorbance was measured at 550 nm with a VICTOR™ X Series Multiple Plate Reader.

#### Confocal microscopy

Biological studies were conducted on cell line NHDF (Normal Human Dermal Fibroblasts). The composite material with diameter equal to 2 cm was UV-sterilized and then it was placed into a cell culture incubator. One each sample, a cell suspension volume of 200 µl (∼2 × 10^5^ cells) was applied, and then placed into incubator for 1,5 h at a temperature of 37 °C. After 1.5 h in the incubator 3 ml of culture medium was added to each sample. The samples were then incubated for 96 h at 37 °C. The tests were performed on three samples, for each fiber type. In order to determine the cells presence and their adhesion to the surface of the nanofibers the samples were removed from the incubators, after 96 h of culture, then placed onto a glass slides and fixed with ethyl alcohol. After fixation the samples were stained with 40 µg/ml of propidium iodide to visualize the DNA in the cells. The excess of propidium iodide was removed by washing the samples with distilled water. Later each sample surface was covered with glycerol and then covered with a coverslip. The prepared samples were inspected by confocal microscopy (600 nm wavelength).

#### Evaluation of antimicrobial activity

Antimicrobial properties of prepared nanofibers were tested on standard microbial agents *S. aureus* ATCC 25923, *C. albicans* ATCC 10231, and *E. coli* ATCC 25922. All tests were performed in quadiplicates. Square (10 × 10 mm) plasma-sterilized samples of the tested nanofibres were immersed in 4 ml of microbial solution 1.5 × 10^5^ CFU/ml, incubated for 17 h at 37 °C, under atmospheric oxygen. Then, 20 µl of medium samples from each incubation were seeded on a solid growth support (Bacto-agar for the bacteria, or Sabouraud-agar for *C. albicans*), and incubated for further 48 h at 37 °C. A total of 20 µl of microbe-free medium samples were used as negative controls, whereas the same quantities of microbial cultures, that were not in contact with the tested nanofibers, were used as positive controls. After 48 h of incubation, the anti-fungal efficacy (AFE), or anti-bacterial efficacy (ABE) were calculated according to the following formula:

\begin{eqnarray*}\mathrm{AFE}[]= \frac{{V}_{c}-{V}_{t}}{{V}_{c}} 100 \end{eqnarray*}

where: *V*_c_, microbial growth density by positive control; *V*_t_, microbial growth density in tested sample. The obtained data is presented in Table 1.

**Figure 2:**
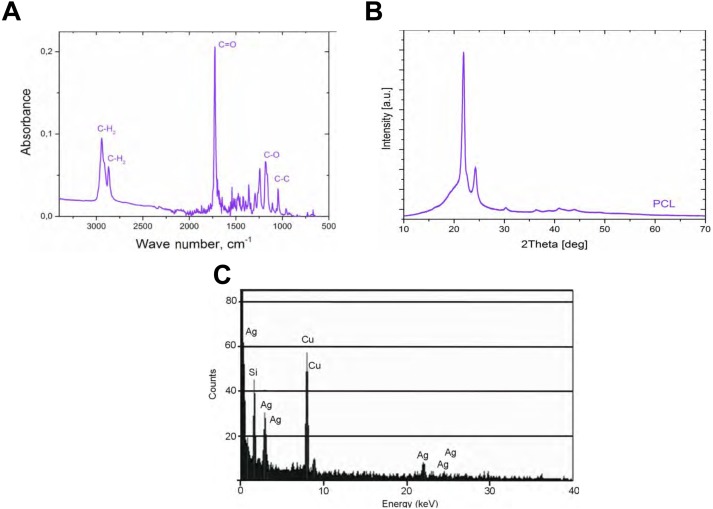
The analysis of the obtained with our process PCL nanofiber. FTIR for polycaprolactone nanofiber (A), XRD spectrum for nanofiber polycaprolactone (B), and EDS for silver nanoparticles (C).

**Table 1:** Antimicrobial efficacy (AME) against the *Staphylococcus aureus*, *Escherichia coli*, and *Candida albicans*. Medians of antimicrobial efficacy (AME) against the *Staphylococcus aureus* ATCC 25923, *Escherichia coli* ATCC 25922 and *Candida albicans* ATCC 10231 standard strains for the PCL nanofibers with different concentrations of silver nanoparticles, and Kruskal–Wallis tests results (*α* = 0.05).

Material	Antimicrobial efficacy (AME), %					
	*Staphylococcus aureus* ATCC 25923 (*p* = 0.003)^‡^		*Escherichia coli* ATCC 25922 (*p* = 0.012)^‡^		*Candida albicans* ATCC 10231 (*p* = 0.016)^‡^	
	Median	Min/Max	Median	Min/Max	Median	Min/Max
PCL0/PCL0 (*p* = 1)^†^	0	^*^	0	^*^	0	^*^
PCL0/PCL_AG_ (*p* = 0.437)^†^	99.9	^*^	99.9	55/99.9	99.9	**0/99.9**

**Notes.**

†*P*-values refer to the differences of AME listed in rows (different standard strains for a particular material) and

‡refer to differences of AME listed in columns (diverse antimicrobial agent concentrations after standard strain).

* no changes in quadruplicate

# both were above 99.9 but less than 100.

## Results

**Figure 3:**
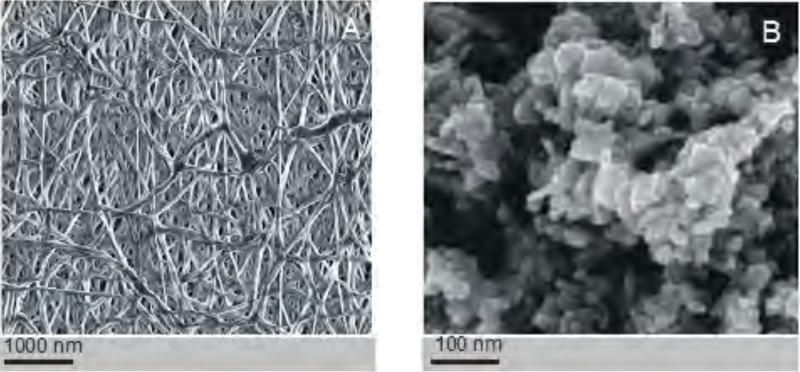
Assessment of the electrospinned nanofibres. The structure of the polycaprolactone nanofibers (no dopant added) (A). The structure of silver nanoparticles used as a dopant in some coaxial fibres (B). Images taken using a scanning electron microscope (SEM).

**Figure 4:**
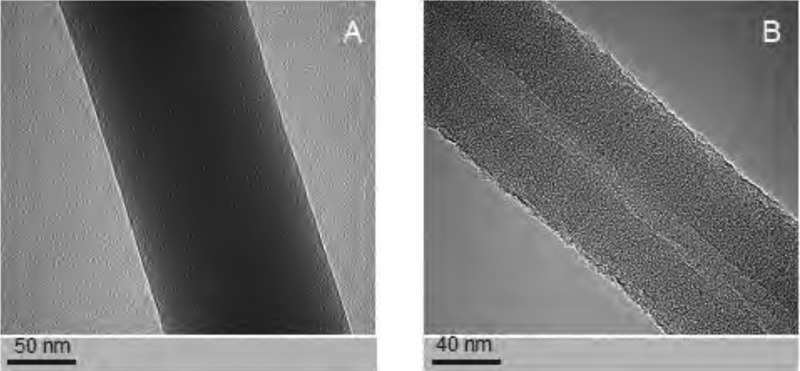
Examples of the nanofibres generated by electrospin, as assessed by TEM. The structure PCL10 nanofiber obtained with a standard electrostatic field electrospin (A). The core-shell PCL5/PCL10 nanofibers obtained with coaxial electrospinning (B). Photographs taken using a transmission electron microscope (TEM).

**Figure 5:**
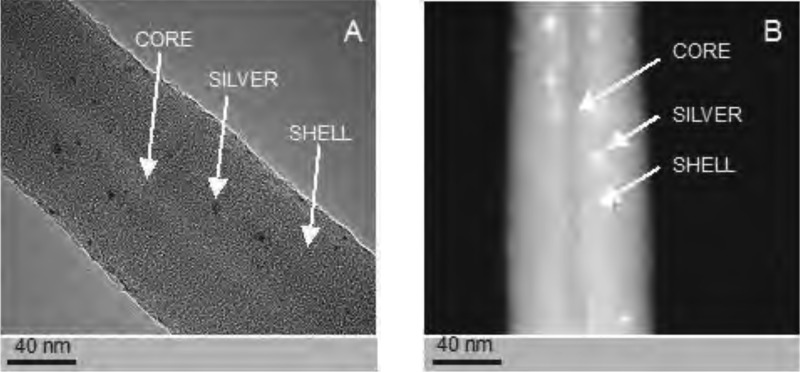
Examples of silver-nanoparticle-doped nanofibres generated by electrospin. The structure of the core-shell PCL_5_/PCL_Ag_ nanofibers observed in the TEM (BF mode) (A). The structure of the core-shell PCL_5_/PCL_Ag_ nanofibers observed in TEM (HAADF-STEM mode) (B). Images of silver nanoparticles are clearly visible in both pictures.

The PCL nanofibers obtained by electrospin from its 10% solution in a mixture of formic acid and acetic acid were analyzed by the spectrometer FTIR (Fig. 2). The analysis confirmed the presence of: (i) stretching vibrations of C = O for the wave 1,720 cm^−1^, (ii) symmetrical stretching vibration of C-H_2_ of wave 2,866 cm^−1^, (iii) asymmetrical stretching vibration C-H_2_ wave 2,943 cm^−1^, (iv) the stretching vibration of C-O and C-C for the wave 1,294 cm^−1^, and (v) the stretching vibration of C-O and C-C to wave 1,163 cm^−1^, characteristic for PCL. Using scanning electron microscopy, SEM (Fig. 3A), and TEM transmission electron microscopy (Fig. 4A) confirmed the presence of PCL nanofibers in the test samples, and provided the valuable information about their morphology. The use of a combination of formic acid with good electrical conductivity and acetic acid with low electrical conductivity in a proportion of 70:30 m/m as a starting PCL solvents, proved to be a successful solution allowing for stabilization of the electrospinning process of nanofibers. It also allowed for obtaining a core-shell nanofibers with the diameter of less than 100 nm. (Fig. 4B, and Fig. 5A, Fig. 5B). The core and shell components are clearly visible both due to differential density of the respective forming materials, and also due to their different thickness. While the same material (PCL) was used for both the core and the shell formation, its starting concentrations were different. The differences in initial concentrations of used PCL solutions effected in different evaporation rates and effected in the final differential density of formed core and shell.

**Figure 6:**
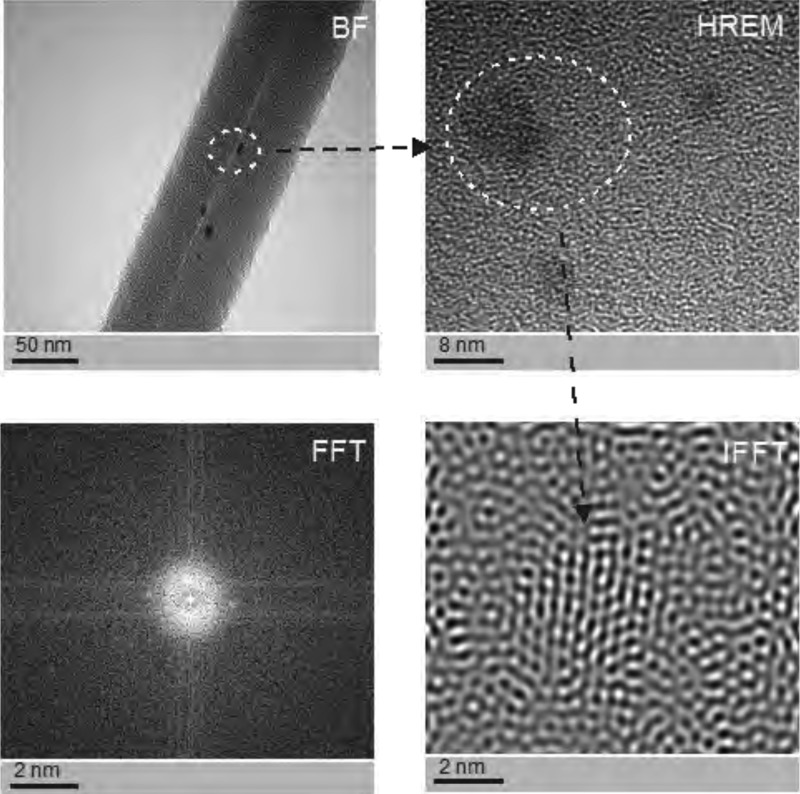
The structure of silver nanoparticles used to generate the doped coaxial nanofibres. Photographs were taken using a high resolution TEM in Fourier Transform mode (FFT), inverse Fourier transform mode (IFFT), the BF mode, and HREM mode. See individual reflections silver FFT correspond to the planes (200), i.e., the FFC silver nanostructres.

SEM-based investigation of obtained nanofibers (Fig. 3B) made it possible to determine the grain structure of the used silver nanoparticles. The presence of silver was also confirmed by studies using EDS diffractometer (Fig. 5). The data showed the presence of the plane (200) characteristic of silver. In order of further confirm the components of these nanoparticles a high-resolution TEM image from the selected area of Fig. 6 was shown in figure 6IFFT and 6FFT. The lattice fringe of 0.205 nm corresponds to the (200) plane of cubic silver.

PCL-ray diffraction studies (Fig. 2) enabled, in turn, to determine the occurrence of two strong peaks 2Θ for 22 °C and 24 °C, which correspond to the reflexes (110) and (200) typical for PCL.

Studies using TEM in bright-field (BF) mode, confirmed the presence of nanofibers of solid-type PCL_10_ (Fig. 4A), coaxial nanofibers PCL_5_/PCL_10_ (Fig. 4B), and PCL_5_/PCL_Ag_ (Fig. 5A TEM BF-mode and Fig. 5B TEM STEM-HAADF-mode).

PCL_10_ fibers, coaxial PCL5/PCL_10_-, and PCL_5_/PCL_Ag_ fibers, all are characterized by regularity in diameter and lack of surface defects such as so-called beads. Hence, this shows that the combination of solvents used, and the chosen molecular weight of the polymer, allow for reliable production of nanofibers, through a very stable electrospinning process.

Using TEM, we estimated the diameter of the PCL_10_ nanofibers to be 98 ± 5 nm, the diameter of the obtained coaxial filaments PCL_5_/PCL_10_ to be 93 ± 5 nm, whereas the diameter of the silver-nanoparticle-doped coaxial nanofibers PCL_5_/PCL_Ag_ to be 86 ± 5 nm. Interestingly, we have observed the decrease in the diameter of the coaxial nanofiber PCL_5_/PCL_10_ as compared to solid nanofibers PCL_10_, and a further decline in fiber diameter for the silver-nanoparticle doped coaxial nanofibres PCL_5_/PCL_Ag_ as compared to coaxial nanofibers PCL_5_/PCL_10_ that do not contain the silver nanoparticles.

The diameter of the core of our coaxial nanofiber PCL_5_/PCL_10_, is 15 ± 3 nm, and it represents 17–19% of the total diameter of the fiber. Whereas for the silver-nanoparticle doped nanofibres PCL_5_/PCL_Ag_, the diameter is 12 ± 3 nm, thus representing 14–16% of the total diameter of the fiber. Thus, the thinning of the silver-doped nanofibers was mainly due to the thinner core produced by the electrospinning process. Importantly, in both types of coaxial nanofibers, the cores occupy central part of the fiber. This shows that the electrospinning produced high-quality nanofibers (the thickness of the coating evenly distributed around the core).

**Figure 7:**
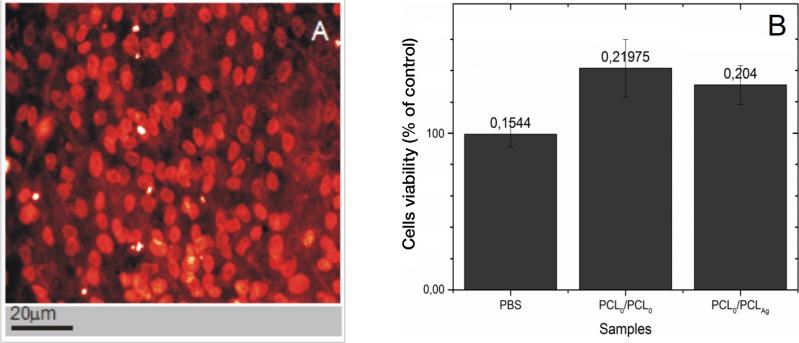
PCL_5_/PCL_10_ are nontoxic, and the support well growth of human cells. An example of cell growth on PCL5/PCL10: confocal microscopy micrographt of NHDF cells stained with propidium iodide (DNA) that were grown for 96 h on the nanofibers PCL5/PCL10 (A). The evaluation of NHDF cell survival by MTT assays (B).

The biocompatibility (potential cytotoxicity) of the produced coaxial nanofibers was assessed using propidium iodide staining of cell nuclei and subsequent observation under confocal microscopy (assessment of shrunken, brightly-stained, apoptotic nuclei, Fig. 7A), and by MTT assay (detection of mitochondrial oxidation, Fig. 7B). The microscopic assessment of cells stained with propidium iodide indicate that the frequency of dead cells with condensed (brightly-stained) nuclei was below 10%, hence consistent with naturally-occurring cell death in culture. The data show that both types of nanofibers (PCL_5_/PCL_10_ and PCL_5_/PCL_Ag_) exhibit good biocompatibility, as assessed using NHDF cells. Surfaces of the tested coaxial nanofiber PCL_5_/PCL_10_ and PCL_5_/PCL_Ag_ are not toxic to the cells and allow them for attachment and metabolic activity. While more advanced tests are necessary, the preliminary data indicate that the structure of the obtained filaments supports NHDF cell proliferation, thus these materials should be subjected to *in vivo* assays to assess their potential use as a tissue scaffold replacements. The observed in MTT-assay apparent increase of cell viability on nanofibers is likely due to the increased surface area offered to the growth of tested fibroblasts. Since MTT-assay measures *de facto* mitochondrial oxidative activity, the available increase of space likely allows for more mitochondria per cell and in effect higher values in MTT-assay.

Finally, we have checked the antimicrobial potential of our nanofibers by using model microbes *Staphylococcus aureus* strain ATCC 25923, *Escherichia coli* strain ATCC 25922, and *Candida albicans* strain ATCC 10231. As shown in Table 1, in contrast to the PCL0/PCL0, that as expected, were non-toxic to the tested microorganisms, the PCL0/PCL_AG_ killed most of *S. aureus, C. albicans,* and *E. coli.* The presented data clearly underlines the antibacterial activity of nano-silver incorporated into our PCL fibers.

## Discussion

Hereby we describe the production and testing of electrospinned (coaxial) nanofibers; some doped with Ag-nanoparticles. Instead of a highly toxic organic solvent normally applied, a less toxic organic-acids were employed to dilute the polymers used for electrospin. Our study describe how to obtain core-shell nanofibers in an electrostatic field with a 10% solution of the polymer dissolved in a mixture of formic acid and acetic acid in a ratio of 70:30 m/m. Biomaterials which are produced to substitute natural tissue scaffolds should meet certain criteria, among others, lack of toxicity, exhibit biocompatibility, sufficient strength, the optimal porosity and surface available for cell adhesion (O’Brien, 2011). Compliance with these criteria is a necessary condition for the biomaterial to functionally restore the natural structure of the tissue.

The lack of toxicity of the electrospinned scaffolds depends on the properties of the starting materials from which it was made, and also to the lesser degree, on the used solvents (traces of solvents usually are difficult to be removed form the biomaterial), it also depends on possible contaminants, i.e., metal ions that may be toxic (Hashemi et al., 2007). The literature frequently describes DMF (dimethylformamide), THF (tetrahydrofuran), chloroform, or methanol as solvents used for PCL tested in this work (Andrady, 2008; Khajavi & Abbasipour, 2012). These organic solvents are highly toxic to cells, and their residual amounts may leak from the implanted into the body biomaterial. This issue is typically ignored by the authors when discussing biocompatibility, and lack of toxicity of the electrospun biomaterials, mostly because such assessments are based either on nanofiber extracts tested for cytotoxicity, and/or on a short *in vitro* study, typically lasting a few days. Under such conditions the residual toxicity of the used organic solvents would not be fully revealed. Such *in vitro* studies will unlikely reveal residual toxicity of organic solvents, because the cells are in contact only with surface of the tested fibres, that usually would be solvent-free (evaporated), and the tested material is immersed in large quantities of cell medium which further minimizes solvents’ potential toxicity. Upon implantation, the conditions differ, because as PCL degrades (complete degradation of PCL may take up to two years) it releases the residual organic solvents. Hence, the *in vitro* studies are unable unequivocally state whether the material throughout the period of degradation would be a nontoxic to-, and biocompatible with the surrounding cells because trapped in the structure solvents such as chloroform, DMF, or others, may be released during the degradation of the polymer and could adversely affect the surrounding cells.

To avoid the prospective leakage of toxic organic solvents from the electrospun coaxial nanofibers we have dissolved the PCL in a mixture of formic acid and acetic acid. As shown in the Figs. 3A, and 4B the electrospinning process was very stable, and in all three types of experiments it generated smooth nanofibers with a regular diameter. The electrostatic field was responsible for the observed decrease in the coaxial fiber diameter PCL_5_/PCL_10_ as compared to the non-coaxial fiber PCL_10_. The electrostatic field was tuned the way that it would stretch the coating solution and guarantee optimum friction for stretching of the core. The observed further decrease in PCL_5_/PCL_Ag_ fiber diameter as compared to PCL_5_/PCL_10_ fibers corresponds to the presence of silver. Metallic silver is more conductive to electrical charge than popular in the industry copper. Hence, the added silver that was uniformly distributed in the solution by prior ultrasound-homogenization, increases the conductivity of the solution. As shown by us, and by others (Chen & Schluesener, 2008; Dallas, Sharma & Zboril, 2011), doping of nanofibers with silver nanoparticles offers an additional advantage, namely it makes the nanofibers resistant to microbial colonization. Graft infection is a fairly common problem in post-surgery care (Fujii & Watanabe, 2015; Revest et al., 2015). Hence incorporation of silver nanoparticles into bioscaffolds not only improves its electroconductivity but also diminishes the risk of infectious post-operative complications.

Electric conductivity is responsible for stretching the fibers. As already discussed above, the increase of conductivity of the mixture (polymer + solvents) will reduce the diameter of the obtained nanofibers. This is due to the higher stretching of the fibers by the present electrostatic forces. This was clearly revealed by our experimenys since doping of PCL with nanosilver resulted in generation of thinner nanofibers. Viscosity of the electrospinning solution has the opposite effect as electric conductivity. Higher viscosity results in the higher diameter of electrospun fibers, because the electrostatic forces will be less effective in stretching the formed nanofibers. Hence, the final result of the electrospining process (either nanofibres or microfibers) is determined by competing forces in the solution caused by electrostatic and viscosity properties.

The applied in our experiments mixture of the solvents is very well suited for biological use as they have a much lower toxicity, when compared to commonly-described in scientific papers organic solvents such as THF, DFM, chloroform, or methanol. Both solvents that we used could easily enter basic cellular metabolic pathways (Lecca, 2014). The FTIR studies were conducted to determine structure and integrity of the obtained PCL fibers. The FTIR was conducted using dry PCL fibers. FTIR studies have confirmed the presence and integrity of PCL, hence, the used combination of solvents did not adversely affect the surface of the obtained polymers. Lack of toxicity of the resulting tissue scaffold-replacement was confirmed by the MTT assays. The prepared by us biomaterials could potentially be used in surgery. The described new materials form a good substrate as experimental tissue scaffolds for preparation of experimental artificial organs by using both induced-pluripotent stem cells (iPS) and also transdifferentiation techniques (Cieslar-Pobuda et al., 2016; Cieslar-Pobuda et al., 2015; Gelmi et al., 2016). Their coaxial structure allows for embedding in the core medicinally-active substances. *In vivo* studies are necessary to further confirm the biocompatibility of the prepared nano-biomaterials.

The development of biomaterial-based artificial tissues offers protection from an accidental transfer of viral infections (marked risk in case of human- or animal-derived donor tissues). Viruses may contribute to carcinogenesis; such as, for example, human papillomaviruses (cervical and oral cancer) (Liu et al., 2016; Zaravinos, 2014), Human T-cell Leukemia Virus (hairy cell leukemia) (Chlichlia et al., 2002; Los et al., 1998) and so on. However, as with some other diseases (Likus et al., 2016c), it is sometimes difficult to clearly associate the given pathogen with cancer etiology. On the other hand, many viruses preferentially, or exclusively replicate in dividing cells; hence, their components are potentially becoming an important source/inspiration in the search of drugs that preferentially target cancer stem cells (Akbari-Birgani et al., 2016; Farahani et al., 2014; Jain et al., 2015; Jain et al., 2016). Beside viral methods, some clinically tested drugs as well as new drugs with preferential toxicity towards cancer stem cells are increasingly becoming available (Jangamreddy et al., 2013; Likus et al., 2016a; Likus et al., 2016b; Moghadam et al., 2015; Moosavi et al., 2016). Such tasks are facilitated by recent progress in methodologies that allows for better detection and monitoring of cancer stem cells (Cieslar-Pobuda et al., 2016; Cieslar-Pobuda et al., 2015; Likus et al., 2016b).

##  Supplemental Information

10.7717/peerj.4125/supp-1 Supplemental Information 1 Data for Fig. 7B

## Abbreviations

BF: Bright-Field
ECM: extracellular matrix
EDS: Energy Dispersive
DMF: Dimethylformamide
FTIR: Fourier Transform Infrared Spectroscopy
HAADF: High-Angle Annular Dark-Field
IFFT: Inverse Fast Fourier Transform
KBr: Potassium Bromide
N6: Nylon 6
NHDF: Normal Human Dermal Fibroblasts
m/m: masse to masse
PCL: Polycaprolactone
PCL_5_/PCL_10_: 5% polycaprolactone/10% polycaprolactone
PCL_5_/PCL_Ag_: 5% polycaprolactone/polycaprolacone with silver nanparticles
PCL_10_: 10% polycaprolactone
PMMA: Poly(methyl methacrylate)
PVA: Polyvinyl Alcohol
SEM: Scanning Electron Microscope
STEM: Scanning Transmission Electron Microscopy
TEM: Transmission Electron Microscopy
THF: Tetrahydrofuran
XRD: X-ray Diffraction

## Additional Information and Declarations

### Data Availability

The following information was supplied regarding data availability: Data for Fig. 7B has been uploaded as a Supplementary File, and other data is included in the manuscript in the form of electron micrographs.
